# Targeting Colorectal Cancer Proliferation, Stemness and Metastatic Potential Using *Brassicaceae* Extracts Enriched in Isothiocyanates: A 3D Cell Model-Based Study

**DOI:** 10.3390/nu9040368

**Published:** 2017-04-10

**Authors:** Lucília P. Pereira, Patrícia Silva, Marlene Duarte, Liliana Rodrigues, Catarina M. M. Duarte, Cristina Albuquerque, Ana Teresa Serra

**Affiliations:** 1 iBET, Instituto de Biologia Experimental e Tecnológica, Apartado 12, 2780-901 Oeiras, Portugal; lu.pebre.pereira@gmail.com (L.P.P.); liliana.rodrigues@ibet.pt (L.R.); cduarte@itqb.unl.pt (C.M.M.D.); 2 Instituto de Tecnologia Química e Biológica António Xavier, Universidade Nova de Lisboa (ITQB NOVA), 2780-157 Oeiras, Portugal; 3 Unidade de Investigação em Patobiologia Molecular (UIPM), Instituto Português de Oncologia de Lisboa Francisco Gentil, E.P.E (IPOLFG, EPE), 1099-023 Lisboa, Portugal; palsilva@ipolisboa.min-saude.pt (P.S.); mrcduarte@ipolisboa.min-saude.pt (M.D.)

**Keywords:** colorectal cancer, cancer stem cells, metastasis, phenethyl isothiocyanate, sulforaphane, *Brassicaceae* extracts, watercress, broccoli

## Abstract

Colorectal cancer (CRC) recurrence is often attributable to circulating tumor cells and/or cancer stem cells (CSCs) that resist to conventional therapies and foster tumor progression. Isothiocyanates (ITCs) derived from *Brassicaceae* vegetables have demonstrated anticancer effects in CRC, however little is known about their effect in CSCs and tumor initiation properties. Here we examined the effect of ITCs-enriched *Brassicaceae* extracts derived from watercress and broccoli in cell proliferation, CSC phenotype and metastasis using a previously developed three-dimensional HT29 cell model with CSC-like traits. Both extracts were phytochemically characterized and their antiproliferative effect in HT29 monolayers was explored. Next, we performed cell proliferation assays and flow cytometry analysis in HT29 spheroids treated with watercress and broccoli extracts and respective main ITCs, phenethyl isothiocyanate (PEITC) and sulforaphane (SFN). Soft agar assays and relative quantitative expression analysis of stemness markers and Wnt/β-catenin signaling players were performed to evaluate the effect of these phytochemicals in stemness and metastasis. Our results showed that both *Brassicaceae* extracts and ITCs exert antiproliferative effects in HT29 spheroids, arresting cell cycle at G_2_/M, possibly due to ITC-induced DNA damage. Colony formation and expression of LGR5 and CD133 cancer stemness markers were significantly reduced. Only watercress extract and PEITC decreased ALDH1 activity in a dose-dependent manner, as well as β-catenin expression. Our research provides new insights on CRC therapy using ITC-enriched *Brassicaceae* extracts, specially watercress extract, to target CSCs and circulating tumor cells by impairing cell proliferation, ALDH1-mediated chemo-resistance, anoikis evasion, self-renewal and metastatic potential.

## 1. Introduction

Colorectal cancer (CRC) is one of the leading causes of mortality worldwide and its incidence is estimated to increase due to the increasing life expectancy of the world’s population and acquisition of unhealthy western lifestyle habits [1]. In CRC onset and progression, mutations in genes encoding Wnt signaling components (like APC, β-catenin, AXIN2) may result in the deregulation of the Wnt/β-catenin signaling pathway. This leads to the intracellular accumulation of β-catenin that forms a complex with TCF7L2 and activates transcription of tumorigenesis and epithelial-to-mesenchymal transition (EMT) “driver” genes [2].

After acquiring a mesenchymal phenotype that promote their intravasation towards bloodstream, cells must be able to evade anoikis and survive in the absence of anchorage, until reaching the target site and proliferate into the metastatic tumor [3]. Some cells of the pool of circulating tumor cells can detain stem cell-like traits, self-renewal and metastatic abilities, and present enhanced chemoresistance [4]. Indeed, the content of circulating tumor cells has been correlated with an increased risk of metastasis in non-metastatic CRC patients [5] and it has been suggested as a robust predictor of tumor relapse after surgical resection [6]. 

Tumor recurrence can be attributed to cancer stem cells (CSCs) that can prevail even after chemotherapy, hindering CRC eradication and favoring a high incidence of tumor relapse [7,8]. CD133 (Prominin-1) and LGR5 (Leucine-rich repeat-containing G-protein coupled receptor 5) have been considered as putative colorectal CSC markers related with proliferation, invasion, metastasis and chemoresistance [9,10,11]. Additionally, cancer cells can resort to Aldehyde Dehydrogenase (ALDH) activity as a drug resistance mechanism [12]. Therefore, it is imperative to seek new therapeutic strategies targeting essential aspects of CRC tumor onset and progression, namely cells with mesenchymal and/or CSC-like traits and crucial signaling pathways [8]. 

Naturally-occurring dietary compounds have been drawing attention as an appealing strategy to target CRC, including the CSC niche, self-renewal and metabolism [13]. In this context, the intake of cruciferous vegetables of the *Brassicaceae* family (e.g., broccoli and watercress) has been associated to a reduced risk of CRC, due to their high content of glucosinolates [14,15]. To exert their chemopreventive effects, these phytochemicals must be converted into more bioactive products termed isothiocyanates (ITCs) by plant myrosinase and/or gut microbiota activity. Therefore, although being a promising chemopreventive measure, the intake of these bioactive compounds in daily diet and further conversion into ITCs may not be sufficient to attain a therapeutic concentration in vivo to counteract tumor progression, since the biological conversion and absorption of ITCs may be limited by several biological factors [14].

To overcome the limitations of the natural uptake of the bioactive compounds, high-pressure extraction procedures allied to the concept of a “green” technology have been developed to recover the phytochemicals of interest from fruits and vegetables, while priming for minimal alteration of the phytochemicals and their functional properties, high extraction selectivity and use of “generally recognized as safe” solvents, such as supercritical carbon dioxide [16]. In our previous work, we optimized the supercritical fluid extraction (SFE) conditions for the selective isolation of ITCs, namely phenethyl isothiocyanate (PEITC), from watercress using supercritical CO_2_ and the extracts obtained proved to exert in vitro antiproliferative activity in CRC cells [17]. 

Among all ITCs, PEITC and sulforaphane (SFN) have been highlighted not only due to their antiproliferative effects, but also for their ability to induce apoptosis and to impair CRC metastatic spread by reducing cell migration, invasion and cell growth in anchorage-independent conditions [18,19,20,21,22,23,24,25]. Moreover, both ITCs have also proved to exert anticancer effects and target CSCs of several solid tumors [26,27,28,29,30]. However, despite the recognized role of these ITCs in the modulation of several cellular processes in CRC cells related with tumor progression, little is known about the effect of these phytochemicals in CRC stem-like cells. Until now there is only one study reporting PEITC selective targeting against the stem-like side population via ROS generation, in this niche [31]. 

To the best of our knowledge, the anticancer effects of ITC-enriched natural extracts and respective ITCs in CRC with focus mainly in the CSC-like cells have not been investigated so far. In the present study, we explored the chemotherapeutic potential of ITCs-enriched *Brassicaceae* extracts in CRC focusing in the CSC-like cells. For this purpose, supercritical CO_2_ extracts derived from watercress and broccoli were tested in a CRC 3D-cell model derived from HT29 cells and their effect on cell proliferation, cell cycle arrest and targeting of the metastatic and CSC-like phenotype (self-renewal/tumorigenic potential, Wnt/β-catenin signaling, anoikis evasion and drug resistance) was evaluated and compared with the major ITCs, namely PEITC and SFN. The 3D-cell model was previously developed by our group [32], and due to its 3D architecture and establishment of pathophysiological gradients it offers a functional/biological response to treatment that best recapitulates the in vivo tumor behavior and chemotherapeutic response [33]. 

## 2. Materials and Methods

### 2.1. Standard ITCs

PEITC and R,S-Sulforaphane (a racemic mixture containing the R- and I-isomers, hereinafter abbreviated as SFN) were purchased from Sigma-Aldrich (St. Louis, MO, USA) and LKT Laboratories (St. Paul, MN, USA), respectively. Stock solutions were prepared at final concentration in the range of 10–30 mM. PEITC was diluted in ethanol (Sigma-Aldrich, St. Louis, MO, USA) and SFN was diluted in dimethyl sulfoxide (DMSO; Sigma-Aldrich, St. Louis, MO, USA), and stored at 4 °C and −20 °C, respectively. 

### 2.2. Watercress and Broccoli Extracts

Watercress extract was previously obtained from dried samples provided by the Vitacress Portugal, S.A. company (Odemira, Portugal) using supercritical CO_2_ at 25 MPa and 35 °C, after a pre-treatment of humidified raw material (125%, dry basis) during 60 min at 35 °C under atmospheric pressure, as reported in our previous work [17]. Chemical characterization of watercress extract indicated that these extraction conditions allowed the highest recovery yields of PEITC, the main ITC identified in watercress extract. The same procedure was applied to broccoli samples that were purchased in a local market (Oeiras, Portugal). Since broccoli is a raw material that we found to be poorer in ITCs than watercress, a subsequent step of concentration of this extract was necessary. The final extract was concentrated under a nitrogen stream in order to preserve volatile compounds, which resulted in a 16-fold increase in SFN content. Extracts were stored at 4 °C until further use. 

Both extracts were quantified in terms of ITCs by High-Performance Liquid Chromatography with Diode Array Detection (HPLC-DAD), as previously described [17], using an LaChrome Elite (HITACHI, Tokyo, Japan) apparatus equipped with a diode array detector L-2455. Peak detection for PEITC was performed at 284 nm and for SFN at 254 nm. EZChrom Elite software (HITACHI, Tokyo, Japan, version 3.3.2) was used for data acquisition. The extracts’ composition in potentially bioactive ingredients was analyzed by Gas Chromatography-Mass Spectrometry (GC-MS), using a Shimadzu-QP2010 gas chromatograph (Shimadzu Corporation, Kyoto, Japan), under the conditions previously described by our group [17]. Data acquisition and analysis were performed by GC-MS Solutions software (Shimadzu Corporation, Kyoto, Japan, version 2.10) and the main compounds, including ITCs, were identified by comparison with mass spectra from established libraries. Broccoli extract was quantified in terms of µM of the respective main ITC (SFN), by HPLC-DAD, as previously described [17].

### 2.3. Cell Lines and Culture

Caco-2 and HT29 cell lines were obtained from the Deutsche Sammlung von Microorganismen und Zellkulturen (Barunshweig, Germany) and American Type Culture Collection (ATCC, Manassas, VA, USA), respectively. Cells were cultured in RPMI 1640 medium (Gibco, Carlsbad, CA, USA) supplemented with 10% (*v*/*v*) of heat-inactivated sterile filtered Fetal Bovine Serum (FBS; Biowest, Riverside, MO, USA). For Caco-2 cells, additional supplementation was made with 1% (*v*/*v*) of PenStrep (Gibco, Carlsbad, CA, USA). Cells were routinely grown in T-flasks in a humidified atmosphere at 37 °C and 5% CO_2_. 

### 2.4. 3D Cell Culture Using a Stirred-Tank Culture System

CRC spheroids were generated using a stirred-tank bioreactor as described previously [32], with some modifications. Briefly, HT29 cells were inoculated into a 125 mL spinner flask (Corning, NY, USA) in culture medium with 10% (*v*/*v*) FBS, accounting for a cell density of 2.5 × 10^5^ cells/mL. Spinner vessel was placed on a magnetic stirrer under 40 rpm and cell culture was carried out in a humidified atmosphere with 5% CO_2_ at 37 °C, with increasing stirring speed to 50 and 60 rpm at the time-point of 8 and 28 h post-inoculation, respectively. At the 4th day post-inoculation, half of the bioreactor volume was renewed in a daily basis. HT29 spheroids’ morphology and average size were monitored using a Leica DM1RB inverted microscope equipped with a C295 camera (10× magnification) and ImageJ software. Experiments were performed using spheroids collected between days 6 and 7 of culture.

### 2.5. Cytotoxicity Assay in Caco-2 Cell Model

Cytotoxicity of extracts and standard ITCs was assessed using confluent and undifferentiated Caco-2 cells as a model of the human intestinal epithelium, as previously described [17]. Briefly, Caco-2 cells were seeded into 96-well plates at a density of 2 × 10^4^ cells/well and allowed to grow for 7 days, with medium renewal every 48 h. At day 7, cells were incubated with watercress extract and PEITC with concentrations varying from 1.56 to 200 µM and with broccoli extract and SFN between 0.78 and 100 µM, all diluted in culture medium. Cells incubated only with culture medium and cells incubated with the maximum % (*v*/*v*) of solvent used were considered as controls–for PEITC and watercress and broccoli extracts a maximum of 2.0%, 2.7% and 2.9% of ethanol (*v*/*v*) was used, respectively, whereas for SFN it was used a maximum of 1.0% of DMSO (*v*/*v*), to ensure that at the maximum concentrations tested the cytotoxic effects were only dependent on phytochemicals rather than the content of solvent. After 24 h, cells were washed twice with Phosphate Buffered Saline (PBS, Sigma-Aldrich, St. Louis, MO, USA) and cell viability was assessed using CellTiter 96^®^ AQueous One Solution Cell Proliferation Assay (Promega, Madison, WI, USA) containing MTS reagent, according to manufacturer’s instructions. Absorbances were measured at 490 nm using a Spark^®^ 10M Multimode Microplate Reader (Tecan Trading AG, Männedorf, Switzerland) and cell viability was calculated relatively to control with culture medium (without treatment or solvent). Three independent experiments were performed in triplicate. 

### 2.6. Antiproliferative Assay in HT29 Monolayers

Antiproliferative effect of natural extracts and ITCs was evaluated in HT29 cells grown in monolayer, as described elsewhere [17]. Briefly, HT29 cells were seeded into 96-well plates at a density of 1 × 10^4^ cells/well. After 24 h cells were incubated with different concentrations of extracts and ITCs diluted in culture medium, ranging from 0.78 to 100 µM. The same controls used in cytotoxicity assay were considered. For solvent controls, a maximum of 1.0%, 1.4% and 2.9% of ethanol (*v*/*v*) was used as control for PEITC, watercress extract and broccoli extract treatments, respectively, and 1.0% of DMSO (*v*/*v*) was tested as control for SFN treatment, to ensure that the maximum solvent content does not exert any antiproliferative effect. Cell proliferation was measured after 24 h using MTS reagent, as mentioned above. Three independent experiments were performed in triplicate.

### 2.7. Antiproliferative Assay in HT29 Spheroids

Antiproliferative effect of extracts and ITCs was assessed in HT29 spheroids as described elsewhere [34]. Briefly, spheroids were seeded in 96-well plates at a density of 5 spheroids/well and cell viability prior to treatment (time-point = 0 h) was performed using PrestoBlue Cell Viability Reagent (Life Technologies, Carlsbad, CA, USA) according to manufacturer’s instructions using a Microplate Fluorimeter FLx800 (Bioteck Instruments, Winooski, VT, USA) (excitation and emission wavelengths of 580 nm and 595 nm, respectively). Then, spheroids were incubated with watercress extract, PEITC and SFN with concentrations ranging from 12.5 to 200 µM and broccoli extract from 6.25 to 100 µM. Spheroids grown in culture medium and in culture medium with a maximum of 2.0%, 2.7% and 2.9% of ethanol (*v*/*v*) were used as controls for PEITC, watercress extract and broccoli extract treatments, respectively, and with 0.7% of DMSO (*v*/*v*) as control for SFN treatment. After 24 h, spheroids were washed with PBS and cell viability was assessed by MTS reagent as described above. A blank without spheroids was considered for subtraction of the background fluorescence and absorbance in both assessments. Cell viability was calculated relatively to control with culture medium (without treatment or solvent) by the following equation:

Spheroid Viability (%)= Abs 24 h (treated spheroids)FI 0 h (treated spheroids)Average Abs 24 h of controlAverage FI 0 h of control × 100 %

where Abs 24 h is the absorbance of HT29 spheroids at 24 h without treatment/solvent (control) or after treatment with extracts and ITCs (treated spheroids—cell viability at time point 24 h) and FI 0h is the fluorescence intensity of HT29 spheroids without treatment/solvent (control) or after treatment with extracts and ITCs (treated spheroids—cell viability at time point 0 h).

At least three independent experiments were made using three replicates. 

### 2.8. Cell Cycle Arrest Analysis

HT29 spheroids were seeded in 12-well plates at a density of 100 spheroids/well and incubated with extracts and ITCs in culture medium for 24 h. The tested concentration corresponded to the EC_30_ value of each extract obtained in the 3D antiproliferative assay and each ITC was tested at the same concentration of the respective extract (i.e., approximately 83 µM for watercress extract and PEITC, and 40 µM for broccoli extract and SFN). Samples with spheroids incubated in culture medium and in medium with the highest % (*v*/*v*) of the solvent used (1.2% of ethanol *v*/*v* and 0.4% of DMSO *v*/*v*) were considered as controls, to confirm that these solvent contents do not impair cell proliferation. Cell cycle phase distribution was analyzed by flow cytometry, as described previously by our group [35] with some modifications. Briefly, spheroids were collected, centrifuged (10 min, 200 g) and washed with PBS followed by new centrifugation. Then, after spheroids dissociation with 0.25% Trypsin-EDTA (1×) (Gibco, Carlsbad, CA, USA), the volume of cell suspension equivalent to 1 × 10^6^ cells was centrifuged in the abovementioned conditions and the pellet was washed with cold PBS, followed by new centrifugation. Cells were resuspended in 1 mL of staining solution containing 0.05 mg/mL of Propidium Iodide (Sigma-Aldrich, St. Louis, MO, USA), 1.5% (*v*/*v*) of Triton X-100 (Sigma-Aldrich, St. Louis, MO, USA), 0.7 U/mL of DNase/protease-free Ribonuclease A (Thermo Scientific, Waltham, MA, USA) and 0.01 M of NaCl, followed by incubation for 2 h at room temperature in the dark. Samples were kept at 4 °C until further reading. Cells were sorted using a CyFlow Space flow cytometer (Sysmex-Partec, Görlitz, Germany) and data analysis was performed by FlowMax Software (Sysmex-Partec, Görlitz, Germany). Two independent experiments were made in duplicate and 30.000 events/sample were measured. 

HT29 spheroids were seeded in 12-well plates at a density of 100 spheroids/well and incubated with extracts and ITCs in culture medium for 24 h. The tested concentration corresponded to the EC_30_ value of each extract obtained in the 3D antiproliferative assay and each ITC was tested at the same concentration of the respective extract (i.e., approximately 83 µM for watercress extract and PEITC, and 40 µM for broccoli extract and SFN). Samples with spheroids incubated in culture medium and in medium with the highest % (*v*/*v*) of the solvent used (1.2% of ethanol *v*/*v* and 0.4% of DMSO *v*/*v*) were considered as controls, to confirm that these solvent contents do not impair cell proliferation. Cell cycle phase distribution was analyzed by flow cytometry, as described previously by our group [35] with some modifications. Briefly, spheroids were collected, centrifuged (10 min, 200 g) and washed with PBS followed by new centrifugation. Then, after spheroids dissociation with 0.25% Trypsin-EDTA (1×) (Gibco, Carlsbad, CA, USA), the volume of cell suspension equivalent to 1 × 10^6^ cells was centrifuged in the abovementioned conditions and the pellet was washed with cold PBS, followed by new centrifugation. Cells were resuspended in 1 mL of staining solution containing 0.05 mg/mL of Propidium Iodide (Sigma-Aldrich, St. Louis, MO, USA), 1.5% (*v*/*v*) of Triton X-100 (Sigma-Aldrich, St. Louis, MO, USA), 0.7 U/mL of DNase/protease-free Ribonuclease A (Thermo Scientific, Waltham, MA, USA) and 0.01 M of NaCl, followed by incubation for 2 h at room temperature in the dark. Samples were kept at 4 °C until further reading. Cells were sorted using a CyFlow Space flow cytometer (Sysmex-Partec, Görlitz, Germany) and data analysis was performed by FlowMax Software (Sysmex-Partec, Görlitz, Germany). Two independent experiments were made in duplicate and 30.000 events/sample were measured. 

### 2.9. Detection of ALDH1 Activity

HT29 spheroids were seeded in 6-well plates at a density of 50 spheroids/well and incubated with natural extracts and ITCs for 24 h (at 15 µM and 30 µM for broccoli extract and SFN; 30 µM and 60 µM for watercress extract and PEITC). Aggregates incubated in culture medium and in medium with the highest % (*v*/*v*) of the solvent used were considered as controls (0.9% of ethanol *v*/*v* and 0.6% of DMSO *v*/*v*) to ensure that these solvent contents do not influence ALDH1 activity. After 24 h, spheroids were collected, centrifuged, washed with PBS and dissociated as described above. ALDEFLUOR™ Assay Kit (Stem Cell Technologies, Vancouver, BC, Canada) was used to detect ALDH1 activity in spheroid-derived cells according to manufacturer’s instructions. A negative control using diethylaminobenzaldehyde (DEAB; a specific inhibitor of ALDH1 activity) was prepared for each sample to control the fluorescence background. Cells were sorted using CyFlow Space flow cytometer and FlowMax Software (Sysmex-Partec, Görlitz, Germany), by reading 10.000 events/sample at flow rate of 200–350 events/s. Data analysis was performed using Flowing Software (University of Turku, Finland, version 2.5.1) by establishing sorting gates relative to background fluorescence of DEAB-treated samples. ALDH activity was normalized relatively to control without treatment/solvent. Two independent experiments were made in duplicate.

### 2.10. Soft Agar Assay

Cell growth and proliferation under anchorage-independent conditions using soft agar assay were evaluated as described elsewhere [36], with some modifications. Briefly, 6-well plates were coated with 2 mL of a solution with 0.6% (*w*/*v*) sterile low melting-point agarose (Lonza, Basel, Switzerland) in 1× RPMI medium with 10% (*v*/*v*) FBS, prepared by mixing a solution of agarose at 1.2% (*w*/*v*) in distilled water with 2× RPMI medium supplemented with 20% (*v*/*v*) FBS at a ratio of 1:1. Meanwhile, HT29 spheroids were trypsinized, as described above, and cells were counted, centrifuged and resuspended in PBS. Then, top layers were prepared by mixing a solution of agarose 0.3% (*w*/*v*) in PBS with the volume of cell suspension rendering 1000 cells/mL and the volume of extracts or ITCs rendering a concentration of 1, 3 or 5 µM in a final volume of 2 mL/well, being gently layered over the solidified bottom layers after homogenization. Cells without any treatment, cells treated with the highest % (*v*/*v*) of solvent used (0.15% of ethanol *v*/*v* and 0.05% of DMSO *v*/*v*) and cells derived from 2D cell culture without treatment (to compare the efficiency of colony formation between 2D and 3D cell models) were considered as controls.

Cells were cultured for two weeks under standard conditions, supplemented with 150 µL of RPMI medium with 10% (*v*/*v*) FBS 2–3 times/week. Colonies greater than 50 µm were counted visually and photographed under a microscope (10× magnification) using the open sources Microsoft software Micro-Manager (University of California, San Francisco, CA, USA, version 1.4) and ImageJ software (National Institute of Health, Bethesda, MD, USA, version v1.51k). Efficiency of colony formation was calculated relative to control of cells without treatment or solvent. At least two independent experiments were performed in duplicate.

### 2.11. Expression Analysis of Genes Involved in Cell-Cycle, EMT, Cancer Stemness and Wnt/β-Catenin Signaling 

#### 2.11.1. RNA Extraction and Reverse Transcription

Spheroids were seeded at a density of 50 spheroids/well in 6-well plates and treated with extracts and standards diluted in culture medium (broccoli extract and SFN: 20 µM and 50 µM; watercress extract and PEITC: 50 µM and 120 µM, selecting an approximated value of the both extracts’ EC_50_ as the highest dose tested, as performed for other phytochemicals [37]). HT29 spheroids incubated in culture medium with ethanol or DMSO at the same % (*v*/*v*) present in extracts/ITCs were considered as controls (0.7% and 1.6% of ethanol *v*/*v* were used as controls for treatment with the lowest and highest concentration of extracts and PEITC, respectively, whereas 0.4% and 0.5% of DMSO *v*/*v* was used as control for the lowest and highest dose of SFN, respectively). After 24 h, spheroids were centrifuged (5 min, 200 g) and resuspended in RLT buffer (QIAGEN, Hilden, Germany) with 1% (*v*/*v*) of β-mercaptoethanol with further mechanic dissociation (by pipetting and syringe needle-based dissociation). RNA extraction was performed using the RNeasy^®^ Mini Kit (QIAGEN, Hilden, Germany), according to the manufacturer’s instructions. Total RNA samples were reverse transcribed to cDNA using Superscript II Reverse Transcriptase 10.000 U (200 U/µL) in a T3 Thermocycler (Biometra, Göttingen, Germany). All reagents for reverse transcription reaction were purchased in Invitrogen. Briefly, a mix with 600 ng of total RNA, 0.5 µL of random primers at 3 µg/µL and DEPC-treated nuclease-free water was prepared for each condition towards a final volume of 7.75 µL. After an incubation of 10 min at 70 °C, 12.25 µL of a master mix with 4 µL of 5× First Strand Buffer, 4 µL of deoxynucleotides, 2 µL of DTT (0.1 M), 0.75 µL of RNase Out Recombinant Ribonuclease inhibitor 5.000 U (40 U/µL), 1 µL of reverse transcriptase and 0.5 µL of DEPC-treated nuclease-free water, were added per reaction. Reverse transcription was performed at 42 °C for 1 h and stopped by incubation at 70 °C for 10 min.

#### 2.11.2. Real-Time Polymerase Chain Reaction (qPCR)

qPCR reactions were performed in 96-well plates using an ABI PRISM 7000 Sequence Detection System and SDS Software (both from Applied Biosystems, Foster City, CA, USA) for determination of the threshold cycle (Ct) value. All reactions were carried out in triplicates towards a final volume of 15 µL containing 2 µl of cDNA. For *GAPDH*, *CDH1* (coding for E-cadherin), *CTNNB1* (coding for β-catenin), *AXIN2* and *TCF7L2* genes, reactions were performed using SYBR^®^ Green PCR Master Mix (Applied Biosystems, Foster City, CA, USA). For *PROM1* (coding for CD133), *LGR5*, *VIM* (coding for Vimentin), *CDKN1A* (coding for p21), *CCNA2* (coding for Cyclin A2) genes, reactions were performed using KAPA SYBR FAST qPCR Master Mix (2×) and KAPA SYBR FAST Rox High (50×) (both from Kapa Biosystems, Wilmington, MD, USA). Primer sequences and concentrations used are available upon request. Cycling conditions applied were: 10 min at 95 °C, 40 cycles at 95 °C for 15 s and 60 °C for 1 min. For data analysis, the expression of each target gene was normalized to the corresponding housekeeping gene levels (*GAPDH*). The overall gene expression variation was assessed by the comparative Ct (2^−ΔΔCT^) method. At least two independent experiments were performed in triplicate for the EC_50_ doses and one experiment was made for the doses below the extracts’ EC_50_ at least in triplicate.

### 2.12. Statistical Analysis

Results were expressed as mean ± SD. Statistical analysis of the results was performed using GraphPad Prism 6 software (GraphPad Software, Inc., La Jolla, CA, USA). For cytotoxicity and antiproliferative assays, the IC_50_ and EC_50_ values, respectively, for each sample were calculated using the former software. Comparisons between samples were made by One-way ANOVA analysis, whereas comparisons with more than two variables were performed by a Two-way ANOVA analysis, both following Tukey’s multiple comparison test. Values of *p <* 0.05 were considered as statistically significant. 

## 3. Results and Discussion

### 3.1. Characterization of Brassicaceae Extracts: Phytochemical Composition and Antiproliferative Effect

**Figure 1:**
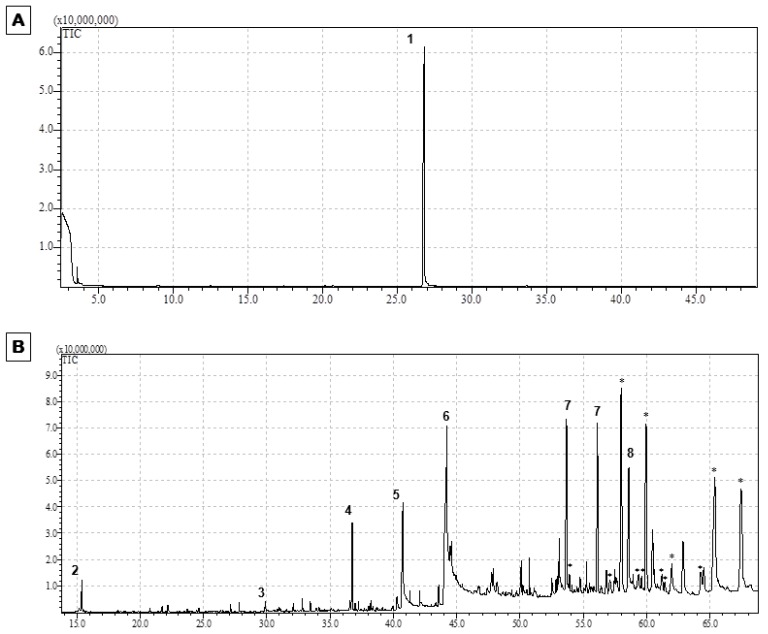
GC-MS chromatographic profiles of potential bioactive compounds present in *Brassicaceae* extracts obtained by supercritical CO_2_ extraction. (**A**) Chromatographic profile of watercress extract; (**B**) Chromatographic profile of broccoli extract; legend: (1) PEITC; (2) 3-Butenyl isothiocyanate; (3) β-PEITC; (4) SFN; (5) I-(+)-Ascorbic acid 2,6-dihexadecanoate; (6) Ethyl Linoleolate; (7) Tetracontane; (8) 1-Eicosanol; (*) compounds without correspondence in the GC-MS library. (Note: Watercress and broccoli extracts were analyzed at a concentration of 7.3 mM PEITC and 3.4 mM SFN, respectively, which were determined by HPLC-DAD, as described previously [17]).

In a previous work, we demonstrated the applicability of SFE process to isolate PEITC-enriched fractions from watercress. Among all extraction conditions, the use of supercritical CO_2_ at 25 MPa and 35 °C applied to freeze-dried watercress, pretreated with 125% of water (dry basis) for 60 min at 35 °C and atmospheric pressure, promoted the highest extraction yield in terms of ITCs (31.7 ± 1.6 µmol ITCs/g dry weight; 29.3 ± 2.6 µmol PEITC/g dry weight). PEITC was the major bioactive compound present in the extract, being identified as the main compound responsible for the antiproliferative effect in HT29 cells [17]. In the present work, the same extraction conditions were also applied to broccoli, aiming at recovering ITCs, such as SFN, since this *Brassicaceae* vegetable is recognized to be a rich source of glucoraphanin, the main precursor of SFN [38]. Although lower extraction yields were obtained for this raw material (1.2 ± 0.2 µmol SFN/g dry weight), this effect could be explained by the lowest content of this ITC in broccoli samples [39] when compared with PEITC content in watercress [17]. Results showed that, while PEITC was the main ITC identified in watercress extract (Figure 1A), GC-MS chromatographic profile revealed that broccoli extract presents a more complex composition in phytochemicals (Figure 1B), including, not only ITCs, but also several fatty acid derivatives. The main ITCs identified included 3-Butenyl isothiocyanate, β-PEITC and SFN, the latter to a greater extent. The presence of fatty acid derivatives, namely vitamin derivatives (I-(+)-ascorbic acid 2,6-dihexadecanoate, derivative of Vitamin C), ester compounds (Ethyl Linoleolate, derivative of linoleic acid) and fatty alcohols (1-Eicosanol) is supported by a previous study focused on obtaining extracts enriched in fatty acids by supercritical fluid technology from broccoli leaves, in which one of the main fatty acids identified was linoleic acid [40]. 

**Figure 2:**
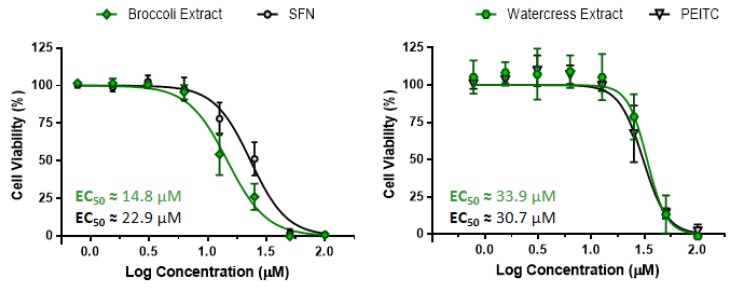
*Brassicaceae* natural extracts and ITCs exerted antiproliferative effects in a dose-dependent manner after exposition for 24 h. Dose-response profiles of the antiproliferative effect induced by *Brassicaceae* extracts and respective ITCs in HT29 cell monolayers used as a 2D cell model of CRC. Results are means of three independent experiments performed in triplicate ± SD. Green: *Brassicaceae* extracts. Black: standard ITCs.

The antiproliferative effect of both extracts was then evaluated using HT29 cell monolayers and the results were compared with the major ITCs identified (PEITC for watercress and SFN for broccoli). Results showed that both extracts and compounds had the ability to impair cell proliferation in a dose-dependent manner, as shown by the dose-response profiles of HT29 cells (Figure 2). Broccoli extract was more effective than SFN (effective concentration values): EC_50_ = 14.8 ± 2.5 µM SFN and EC_50_ = 22.9 ± 3.4 µM SFN, respectively), whereas watercress extract and PEITC displayed similar antiproliferative effect (EC_50_ = 33.9 ± 3.7 µM PEITC and EC_50_ = 30.7 ± 4.8 µM PEITC, respectively). Our findings are in agreement with the phytochemical characterization of these extracts, since in watercress extract the major compound identified was PEITC, being plausible to assume that the antiproliferative effect of this extract is mainly resultant from this ITC, as previously suggested [17]. On the other hand, the complex plethora of phytochemicals in broccoli extract suggested that the enhanced antiproliferative effect of this extract may arise from a synergy/additive effect between SFN and other compounds, namely minor ITCs (3-Butenyl isothiocyanate and/or β-PEITC) and fatty acid derivatives. Notwithstanding, in both extracts, ITCs were the main contributors for their antiproliferative effect, which is in accordance with the antiproliferative effect of PEITC and SFN reported in several human CRC cell lines [18,19,20,22,23]. Importantly, EC_50_ values for all samples are well below the inhibitory concentration that decreases 50% of cell viability (IC_50_) of confluent and undifferentiated Caco-2 cell monolayers (Table S1, Supplementary Material) [35], a cell model that shares some characteristics with crypt enterocytes being widely used to assess the cytotoxicity effect of chemical and natural compounds on the intestinal function [41,42].

### 3.2. Anticancer Effects of Brassicaceae Extracts and ITCs in 3D Cell Model of CRC

#### 3.2.1. *Brassicaceae* Extracts and ITCs Impair Cell Proliferation in HT29 Spheroids in a Dose-Dependent Manner 

After screening the potential antiproliferative effect of extracts and ITCs in a 2D cell model, we evaluated whether these samples were capable to decrease cell proliferation in a more complex biological scenario, resorting to our previously optimized 3D cell model of HT29 cell spheroids (Figure S1, Supplementary Material) that reach an average diameter of 500 µm at the 7th day of culture in stirred conditions and present an inner necrotic core and hypoxic region, as previously reported [32]. 

**Figure 3:**
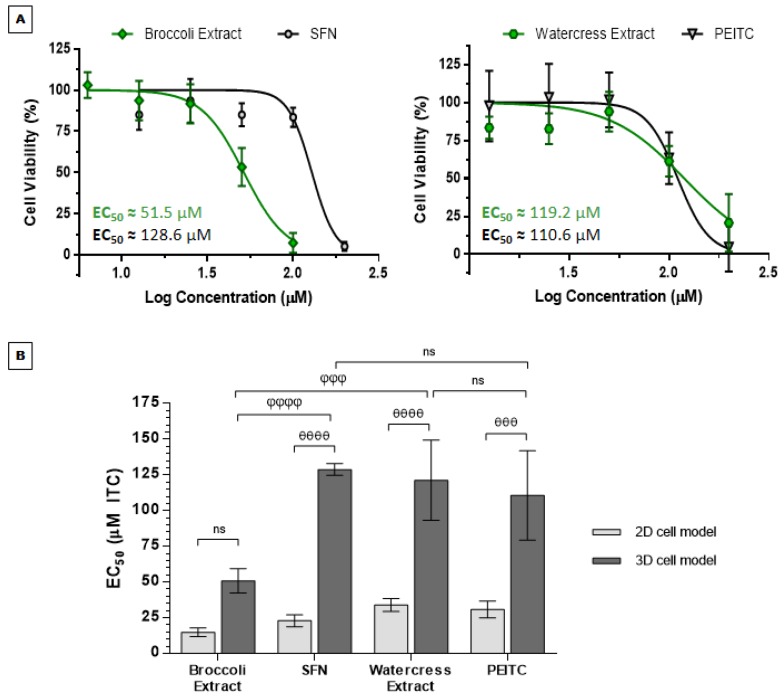
Treatment with natural *Brassicaceae* extracts and ITCs impair cell proliferation in HT29 spheroids after exposition for 24 h. (**A**) Dose-response curves of the antiproliferative effect induced by broccoli and watercress extracts and their main ITCs, SFN and PEITC (left and right panel, respectively). Green: *Brassicaceae* extracts; black: ITCs; (**B**) EC_50_ values of the antiproliferative response induced by *Brassicaceae* extracts and ITCs (presented in µM of ITC) in HT29 monolayers (2D cell model, light grey) and HT29 spheroids (3D cell model, dark grey). Results shown are means of at least 3 independent experiments performed in triplicate ± SD, and a *p <* 0.05 was considered as statistically significant. ^θθθ^ *p <* 0.001 and ^θθθθ^ *p <* 0.0001 are relative to the same extract/ITC between both cell models; ^ϕϕϕ^ *p <* 0.001 and ^ϕϕϕϕ^ *p <* 0.0001 are relative to broccoli extract.

As shown in Figure 3A,B, broccoli extract induced a significantly higher antiproliferative effect in HT29 3D cell model than SFN, as demonstrated by the dissimilar dose-response profiles with EC_50_ values varying about 2.5-fold (*p <* 0.0001). In contrast, cells treated with watercress extract and PEITC displayed a similar antiproliferative response as demonstrated by the dose-response curves and EC_50_ values (Figure 3A,B). These results are in line with those obtained in cell monolayers, confirming that the antiproliferative outcome promoted by watercress extract is mainly attributable to PEITC, whereas in broccoli extract other phytochemicals in addition to SFN may also contribute to the antiproliferative response.

Moreover, to prevent cell proliferation in HT29 spheroids, higher doses of extracts and ITCs were required in comparison with cell monolayers (Figure 3B). In particular, the EC_50_ value for watercress extract and PEITC showed an increase of approximately 4-fold, and broccoli extract and SFN an increase up to almost 3- and 6-fold, respectively. The general need to increase dose treatment to circumvent the limited anticancer compound diffusion and to have the intended biological response in more complex cellular scenarios, was already verified in 3D cell models of breast and hepatic cancer cells [43,44]. 

Our results demonstrated that HT29 cells were more susceptible to treatment when cultured in 2D conformation (monolayers) in contrast to HT29 spheroids that encompass a significantly higher cell density assembled in a 3D cell structure with an average size of 500 µm (Figure S1, Supplementary Material). This is of the outmost importance since, in spheroids that reach this size, a hierarchy of pathophysiological gradients is established, leading to oxygen and nutrient input limitations, as well as to catabolites accumulation, within the spheroid which culminates in the formation of necrotic cores [33,45], as already verified in our previous findings [32]. Therefore, the discrepancy observed in the EC_50_ values of both cells models could be related with phytochemicals penetration in spheroids, since their own 3D architecture could offer phytochemical’s diffusion limitations (due to production of ECM components and/or establishment of tight cellular junctions) and acquisition of a more chemoresistant phenotype [33,43,44,45,46,47,48]. 

#### 3.2.2. *Brassicaceae* Extracts and ITCs Arrested Cell Cycle at G_2_/M Phase in HT29 Spheroids 

**Figure 4:**
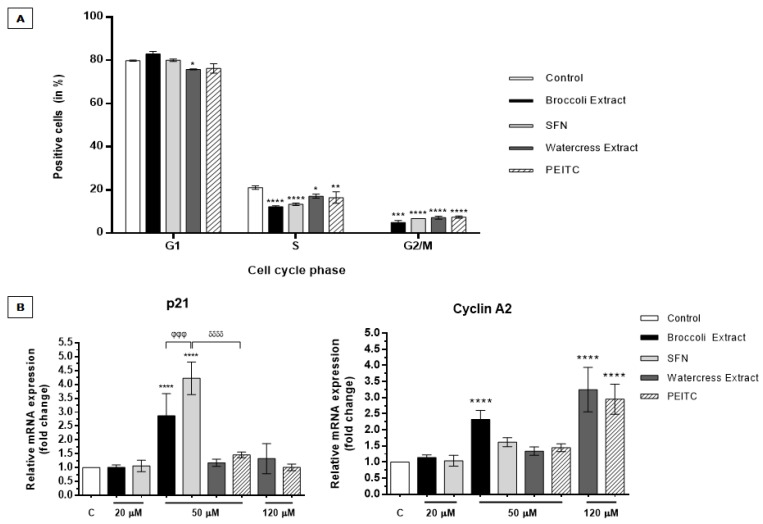
*Brassicaceae* extracts and ITCs impair cell proliferation in HT29 CRC spheroids by arresting cell cycle at G_2_/M phase and deregulating *CDKN1A* and *CCNA2* (coding for p21 and Cyclin A2 markers) levels after exposition for 24 h. (**A**) Cell cycle distribution in HT29 spheroids analyzed by flow cytometry after treatment with broccoli and watercress extracts and respective ITCs. Results are means of two independent experiments performed in duplicate ± SD; (**B**) Analysis of *CDKN1A* and *CCNA2* expression by qPCR in HT29 spheroids normalized relatively to treatment with the same % (*v*/*v*) of solvent used for each extract/compound. *GAPDH* was used as endogenous control. In the case of the extracts’ EC_50_ values (50 and 120 µM for broccoli and watercress extracts, respectively) results are expressed as mean of two independent experiments performed in triplicate ± SD. For the tested dose bellow the EC_50_ value, results are expressed as mean of one experiment performed at least in triplicate ± SD. For both assays a *p <* 0.05 was considered as statistically significant. * *p <* 0.05, ** *p <* 0.01, *** *p <* 0.001 and **** *p <* 0.0001 are relative to control; ^φφφ^ *p <* 0.001 is relative to broccoli extract; ^δδδδ^ *p <* 0.0001 is relative to PEITC.

HT29 spheroids were treated for 24 h with *Brassicaceae* extracts and respective major ITCs to evaluate their effect on cell cycle phase distribution of spheroid-derived cells and to ascertain how they impair cell proliferation even at a dose below the EC_50_. For this purpose, we tested 83 µM PEITC for watercress extract and 40 µM SFN for broccoli extract, since these are concentrations required to reduce 30% cell proliferation (EC_30_) in 3D cell spheroids, using the same strategy as other authors [49]. Results showed that the antiproliferative effect promoted by watercress and broccoli extracts, as well as by PEITC and SFN, correlated with G_2_/M cell cycle arrest, as shown by the higher content of cells in this cell cycle checkpoint when compared to control (Figure 4A). Treatment with both extracts and ITCs led to a shift in the cell cycle profile from G_1_ and S phases towards G_2_/M phase, as shown by the discrete reduction in G_1_-positive cells after treatment with watercress extract (*p <* 0.05) and in S-positive cells after treatment with both extracts and compounds for 24 h, accompanied by the significant increase in G_2_/M-positive cells after exposition to broccoli (*p <* 0.001) and watercress extracts (*p <* 0.0001), as well as to their main ITCs relatively to control (*p <* 0.0001), which was practically destitute from cells in this phase. 

Our results suggested that cell cycle arrest at G_2_/M phase induced by extracts is mainly attributable to their respective ITCs, as shown by the similar content of cells in this phase (no significant differences were observed between extracts and ITCs). Considering PEITC effect, the results differed from the G_1_ cell cycle arrest reported in HT29 monolayers [22]. On the other hand, our findings regarding SFN treatment in HT29 spheroids pointing to G_2_/M arrest are in accordance with those obtained in HT29 monolayers [50]. Therefore, it is plausible that cellular context and cell model conformation, as well as dose treatment in the same cell line might elicit different effects regarding cell cycle arrest. 

For gene expression analysis, the dose tested was an approximated value of the EC_50_ of both extracts (50 and 120 µM for broccoli and watercress extracts, respectively). In addition, watercress extract and PEITC were also tested at 50 µM to compare the effect of all phytochemicals at the same concentration. Attending to a 2.4-fold relation between the maximum doses tested (50 and 120 µM), the same proportion was applied to test a lower dose of SFN and broccoli extract (20 µM). Expression of cell cycle markers [*CDKN1A* (encoding p21) and *CCNA2* (encoding Cyclin A2)] was heterogeneous in response to extracts and compounds. Watercress extract and PEITC did not influence p21 expression even with a dose increase up to an approximated EC_50_ value (120 µM) (Figure 4B). In contrast, treatment with broccoli extract and SFN, up to an approximated concentration of extract EC_50_ value (50 µM), significantly induced p21 upregulation in HT29 spheroids relatively to control (*p <* 0.0001), being this effect more pronounced in SFN treatment. Although p21 overexpression commonly suggests induction of cell cycle arrest at G_1_/S phases [51], p21 has also been implicated in G_2_/M transition in response to DNA damage after oxidative stress. However, Dash and El-Deiry showed that when DNA damage occurs in G_2_/M phase, instead of p21 shuttling towards cell nuclei during S-phase, favoring the assembly of cyclin B1-CDK2 kinase, its activation and, hence, G_2_/M progression, p21 assumes a negative regulatory role, arresting cell cycle at this stage [52]. Indeed, p21 was already been reported to be involved in G_2_/M cell cycle arrest in response to DNA damage, either in a context of wild-type and deficient p53 cells [53,54]. Accordingly, it has been reported that ITCs may induce DNA damage generally by oxidative stress and promote G_2_/M cell cycle arrest in several tumors, namely in colon cancer cells [55,56]. Indeed, CRC cells treated with SFN and PEITC have shown to be prone to induction of DNA damage by these ITCs, as verified by comet assay evaluation, and to cell proliferation impairment by G_2_/M cell cycle arrest [56,57]. 

Our results have also shown that exposition to watercress extract and PEITC, at the EC_50_, induced the overexpression of cyclin A2 in our 3D cell model (*p <* 0.0001) (Figure 4B). The same was observed for broccoli extract treatment (*p <* 0.0001), apparently in a lesser extent. Curiously, no significant difference was attained for SFN treatment at the same dose. Taken together, although results from cyclin A2 analysis (Figure 4B) did not confirm the G_2_/M cell cycle arrest results (Figure 4A), they could be related with the DNA-damaging abilities of both ITCs [56,57,58,59] and with DNA double-strand-breaks repair by homologous recombination [60,61]. In agreement, increased Cyclin A expression levels have previously been shown in HT29 monolayers treated with SFN [50].

However, it is also possible that, though treatment with extracts and ITCs may evoke G_2_/M transient arrest and checkpoint (Figure 4A), some cells could evade this apparent antiproliferative effect after DNA damage repair, following to later stages of cell proliferation as shown by increased levels of cyclin A2 (Figure 4B). Moreover, it was reported that after a 24 h treatment with this ITC, synchronized quiescent HT29 cells were able to re-enter in cell cycle [62]. Attending that spheroids with a mean size of 500 µm encompass not only a necrotic core and a highly proliferative outer layer but also an intermediate inner quiescent layer [33,45], one could not discard that cyclin A2 levels might be related with the re-initiation of the cell cycle by spheroid inner G_0_ quiescent cells after treatment with extracts and ITCs, thus suggesting a more chemoresistant phenotype for this HT29 spheroid layer. Curiously, in our results, SFN was the only compound that not elicited significant cyclin A2 overexpression after a 24 h exposure. 

Nevertheless, cell cycle arrest at G_2_/M stage upon SFN exposition was already reported in HT29 cells in parallel with subsequent increase in Cyclin A expression [50]. Further studies should be carried out to explore dose/time-dependent treatments with all samples to analyze the behavior of spheroid-derived cells in terms of cell cycle arrest.

#### 3.2.3. *Brassicaceae* Extracts and ITCs Induced Dissimilar Effects in ALDH1 Activity and Chemoresistance in HT29 Spheroids

ALDH1 is a cytosolic detoxifying enzyme that endorses cancer cells with resistance to chemotherapy. ALDH1 has been linked to stemness traits and recognized as a putative CSC marker, in which high ALDH1 expression in CRC correlates with reduced patient overall survival and advanced tumor stage, being related with lymph node metastasis [12,63]. 

Hence, we next sought to determine whether natural extracts and ITCs were able to target the CSC-like phenotype and evade cell chemoresistance by modulation of ALDH1 activity even at doses bellow extracts’ EC_50_ values obtained in antiproliferative assays, as described in other studies [64,65]. More precisely, the maximum dose tested for each extract and respective ITC was an approximated value of half the extract’s EC_50_ value (30 µM for SFN/broccoli extract and 60 µM PEITC/watercress extract). Lower concentrations were also tested (15 µM for SFN/broccoli extract and 30 µM watercress extract/PEITC) aiming at evaluating the dose-dependent effect and, also, to compare the effect of all phytochemicals at the same concentration (30 µM). Worth mentioning, in comparison with cell monolayers, our 3D cell model displayed a CSC-like phenotype conferred by a significantly higher ALDH1 activity (*p <* 0.01, Figure S2A, Supplementary Material). Therefore, our 3D cell model encompassing a significant subpopulation of ALDH^+^ cells (*p <* 0.001) might recapitulate best the in vivo tumor chemotherapeutic response. 

**Figure 5:**
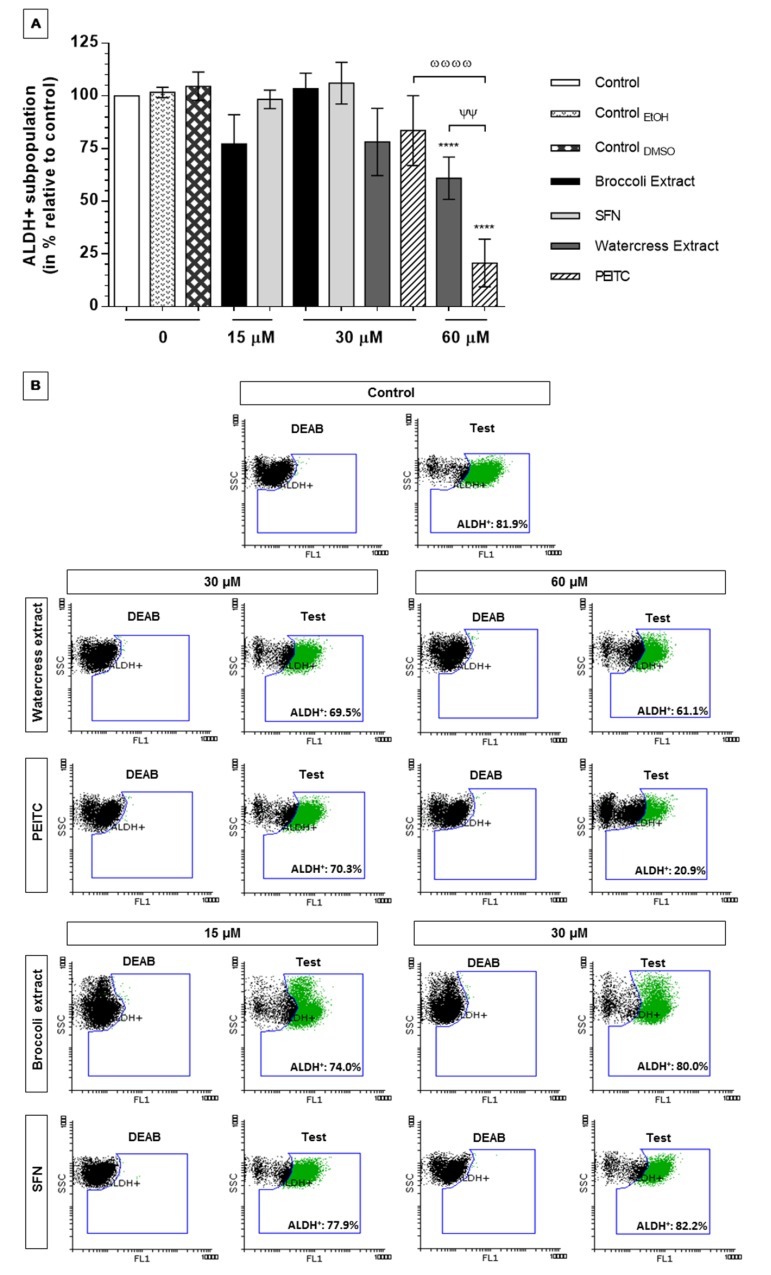
ALDH1 activity in HT29 spheroids after exposition to watercress and broccoli extracts and respective main ITCs, PEITC and SFN, for 24 h assessed by the ALDEFLUOR™ assay. ALDH1 activity was assessed after treatment with submaximal dosages of all phytochemicals, more precisely with ½ and ¼ of extracts’ EC_50_ values obtained in 3D antiproliferative assay. (**A**) Effect of natural extracts and ITCs in ALDH1 activity normalized relatively to control without treatment or solvent (100% ALDH1 activity). Results are means of at least two independent experiments performed in duplicate ± SD; a *p <* 0.05 was considered as statistically significant. **** *p <* 0.0001 is relative to control; ^ѰѰ^ *p <* 0.01 is relative to watercress extract and ^ωωωω^ *p <* 0.0001 is relative to the same compound; (**B**) Representative dot-plots of ALDH1 subpopulations in HT29 spheroids from flow cytometry analysis using ALDEFLUOR™ assay. Negative control was treated with DEAB (ALDH1 inhibitor). The content of ALDH^+^ cells for each representative sample is indicated as % relatively to total cell population.

To the best of our knowledge, there are no reports focusing on SFN and PEITC effect in ALDH1 activity in CRC stem-like cells. Despite the cumulative evidences on SFN effect in ALDH1 activity of cancer stem-like cells of other solid tumors [26,27,28,29], in our experiments the effect of this ITC, either directly or indirectly through the extract, was not verified in the context of HT29 spheroids enriched in stem-like cells. As shown in Figure 5A, in the doses tested, broccoli extract and SFN did not demonstrated potential to impair ALDH1 activity and to target ALDH^+^ cells. This can be verified by the absence of variation in the content of ALDH^+^ subpopulation, with about 80% and 82% of ALDH^+^ cells upon treatment with 30 µM of broccoli extract and SFN, respectively, against near 82% of ALDH^+^ cells in control sample (Figure 5B). Therefore, additional tests should be further performed with different doses of extract and SFN.

Contrary, both watercress extract and PEITC decreased ALDH1 activity in a dose-dependent manner within the submaximal dose range tested (i.e., ½ and ¼ of extracts’ EC_50_ values obtained in HT29 spheroids) (Figure 5A). At the highest concentration tested (60 µM PEITC), hitting the threshold, a significant reduction in ALDH1 activity was observed (*p <* 0.0001). Indeed, this was supported by a decrease in ALDH^+^ subpopulation from nearly a representative 82% to 61% and to 21% ALDH^+^ cells, for 30 and 60 µM PEITC, respectively (Figure 5B). Results showed a more pronounced biological response to PEITC compared to watercress extract, suggesting that other compounds present in this extract may antagonize with PEITC, rendering a lower effect in targeting ALDH^+^ cells. 

The ineffectiveness of SFN and broccoli extract in ALDH1 activity of HT29 spheroids at the highest dose tested could be related with the action of other possible chemoresistance mechanisms. For instance, despite these phytochemicals did not influenced significantly *ABCB1* expression, coding for P-glycoprotein (P-gp; Figure S3, Supplementary Material), our results do not exclude the involvement of this drug efflux pump in chemoresistance. It might be possible that the lower effect of broccoli extract and SFN might be related with the efflux of this ITC, since SFN is a P-gp substrate contrary to PEITC [66]. Taking these findings together, we suggest that watercress extract and PEITC are promising to evade chemoresistance and eradicate CSCs by targeting the ALDH^+^ subpopulation. 

#### 3.2.4. *Brassicaceae* Extracts and ITCs Impair Self-Renewal and Anchorage-Independent Cell Growth Potential

Previous studies have shown that PEITC and SFN can prevent CRC cell migration, an early prerequisite for cell intravasation and tumor metastasis. For instance, PEITC demonstrated to induce anti-metastatic properties by impairing invasion and migration of HT29 cells in a dose-dependent-manner, through the inhibition of MMP-2 and MMP-9 [24]. On the other hand, it was also suggested that SFN may impair hypoxia-induced cell migration in HCT116 cells by downregulating HIF-1α and VEGF expression [25]. 

However, little is known for the role of these ITCs and, more importantly, of ITC-enriched natural extracts in preventing cell survival and proliferation in anchorage-independent conditions, especially in the context of cells with a CSC-like phenotype. To investigate this, we assessed the anchorage-independent cell growth of spheroid-derived cells in a semi-solid agar matrix to mimic some of the crucial steps of metastasis—anoikis evasion and proliferation in the secondary tumor site [3]. It is noteworthy that single cells derived from our 3D cell model of CRC presented higher colony-forming efficiency compared to monolayer-derived cells when grown in a semisolid milieu, which translates the greater anchorage-independent cell growth, metastatic and tumorigenic ability of these cells (Figure S2B, Supplementary Material). Therefore, it is plausible to assume that the phenotype of HT29 spheroid-derived cells resembles more with the one of circulating tumor cells derived from the primary tumor, in a manner that mimics best the cell self-renewal and anchorage-independent cell growth potential inherent to anoikis evasion, metastasis, and most importantly, CSC-like phenotype [3,4,67].

**Figure 6:**
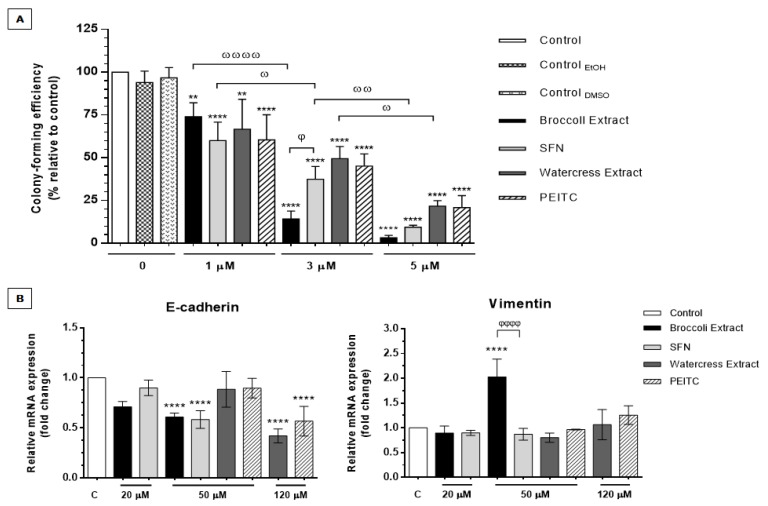
Inhibitory effects of natural extracts and ITCs in anchorage-independent cell growth using cells derived from HT29 spheroids. (**A**) Inhibition of anchorage-independent cell growth upon a 2-week treatment with natural extracts and ITCs normalized relatively to control without treatment or solvent (100% colony formation efficiency). ** *p <* 0.01 and **** *p <* 0.0001 are relative to control; ^ω^ *p <* 0.05, ^ωω^ *p <* 0.01, ^ωωωω^ *p <* 0.0001 are relative to the same compound; ^φ^ *p <* 0.05 is relative to broccoli extract; (**B**) Analysis of *CDH1* and *VIM* expression (coding for E-cadherin and Vimentin, respectively) by qPCR in HT29 spheroids treated with natural extracts and ITCs for 24 h; data normalized relatively to treatment with the same % (*v*/*v*) of solvent used for each extract/compound. *GAPDH* was used as endogenous control. In the case of the extracts’ EC_50_ values (50 and 120 µM for broccoli and watercress extracts, respectively) results are expressed as mean of two independent experiments performed in triplicate ± SD. For the tested dose bellow the EC_50_ value, the results are expressed as mean of one experiment performed at least in triplicate ± SD. **** *p <* 0.0001 is relative to control; ^φφφφ^ *p <* 0.0001 is relative to broccoli extract.

To explore the targeting of stem-like circulating tumor cells by these phytochemicals, single cells derived from HT29 spheroids were treated with extracts and standards and grown in soft agar for 2 weeks, after which colony number and size were evaluated. The concentrations used for this study (1, 3 and 5 µM PEITC/SFN) are lower than the EC_50_ obtained for the extracts and this selection was based on the (i) lower cell density per well and (ii) increased susceptibility to phytochemicals’ exposure of single cells seeded on agar. Importantly, these concentrations are in the range of the tested doses for other bioactive compounds, namely *p*-coumaric acid and tricin, in colon cancer cells [68]. As shown in Figure 6A, all *Brassicaceae* extracts and ITCs inhibited the colony formation capacity of spheroid-derived cells in a dose-dependent manner (*p <* 0.0001). Additionally, all phytochemicals also affected the average colony size as demonstrated for the treatment with SFN and broccoli extract in Figure S4 (Supplementary Material). We demonstrated that these extracts may induce anoikis and impair anchorage-independent cell growth of CRC cells with associated stemness traits, being this effect mainly attributable to ITCs. Therefore, we suggest that ITC-enriched *Brassicaceae* extracts may have the ability to decrease the metastatic and self-renewal potential of colorectal CSCs and/or circulating tumor cells [67].

Consistent with these findings, the increase in Cyclin A2 levels, observed in the present study (Figure 4B), could also be indicative of the anti-metastatic effects of these phytochemicals, since Cyclin A2 interacts directly with RhoA and activates this actin regulator that negatively modulates cell’s motility, invasive and metastatic skills. Depletion of Cyclin A2 has shown to decrease RhoA activity and to modulate the cytoskeletal organization causing a rearrangement in the actin filaments (protrusions) that ultimately enhance cell migration. Moreover, significantly lower levels of this cyclin were found in CRC cells with a more invasive and metastatic phenotype, as well as in metastases compared to primary tumor samples [69]. Therefore, increase in Cyclin A2 levels after treatment with extracts and ITCs might be a predictor of impaired migratory ability in HT29 spheroid-derived cells.

In general, broccoli extract and SFN proved to be the most effective in inhibiting the formation of these second-generation spheroids with the highest concentration tested. In fact, treatment with 5 µM of broccoli extract and SFN almost abolished completely the tumorigenic potential of spheroid-derived cells (3.3% and 9.4% of colony formation efficiency relative to control, respectively) (Figure 6A). Similar results with this ITC had already been reported in a panel of human primary CRC cell lines, in which impairment of anchorage-independent cell growth in vitro was also dose-dependent, and confirmed in vivo [21]. Besides, the effect of SFN in self-renewal, metastatic and tumor initiation skills in oral squamous cell carcinoma and pancreatic cancer stem-like cells were also already reported in vitro and in vivo [26,27]. 

Similarly, treatment with watercress extract and PEITC also induced a significant decrease in the tumorigenic potential in a dose-dependent manner, although it did not lead to such a prominent effect as in the case of broccoli extract and SFN at 5 µM (21.8% and 20.9% of colony formation efficiency relative to control, respectively). Moreover, in a recent report, also PEITC selectivity to stem-like side population was associated with its capacity to modulate the redox status in these cells by increasing their cellular ROS levels, which reduced their growth in vitro and their tumorigenic ability in vivo [31]. Therefore, it is plausible that the ability of watercress extract to target colorectal CSCs and/or circulating tumor cells and to induce anoikis might be related with redox modification.

To further investigate the anti-metastatic effect of extracts and ITCs in HT29 spheroids the expression of EMT-associated markers, *CDH1* (coding for E-cadherin) and *VIM* (coding for Vimentin), was also evaluated. Results showed that both extracts and ITCs promoted E-cadherin downregulation in a dose-dependent manner (Figure 6B), with significant differences attained around the EC_50_ value of both extracts (50 µM and 120 µM for broccoli and watercress extracts, respectively). In a general way, extracts and ITCs did not exert significant effects in Vimentin expression with exception of broccoli extract tested at 50 µM, which was also significantly different from SFN effect at same dose. This might suggest that other(s) compound(s) present in the extract may contribute to this effect, eliciting the overexpression of this mesenchymal marker. Considering the conventional connotation of EMT with a more metastatic phenotype [70,71], our results could suggest that broccoli extract could elicit undesired effects by causing E-cadherin downregulation and overexpression of the mesenchymal marker vimentin. 

However, recent reports have been enlightening the occurrence of a possible paradox, in which the epithelial phenotype attributes to cells a more metastatic behavior, as well as enhanced survival skills [48,72,73]. Indeed, the association between the epithelial phenotype and the generation and/or maintenance of cell spheroids has been proposed. For instance, in spheroids generated with primary tumor CRC cells, E-cadherin was shown to be necessary for the establishment of intercellular interactions between epithelial cells, which secured the maintenance and survival of CRC spheroid cells [73]. Similarly, E-cadherin expression revealed to be required for spheroid formation ability in breast cancer cells grown in suspension, as the knockdown of this epithelial marker in MCF-7 cells impaired their ability to generate mammospheres [74]. Moreover, the maintenance of self-renewal and tumor initiating properties was also associated with E-cadherin expression, and, hence, with an epithelial phenotype. In this context, in cellular models of prostate and bladder cancer cell lines, the induction of EMT in a tumor subpopulation with a marked epithelial signature and tumor initiating properties caused the loss of stemness traits, decreasing anchorage-independent cell growth and metastatic abilities. Indeed, the knockdown of E-cadherin in this epithelial subpopulation with tumor initiating properties impaired spheroid formation in vitro and the ability of these cells to colonize distant organs in vivo [72]. Also, in a 3D model of SKOV-3 ovarian cancer cells it was demonstrated that E-cadherin expression is crucial for spheroid generation and maintenance, and correlates with chemoresistance to cisplatin, abilities that derive from cell-cell adhesion supported by tight cellular junctions. Notwithstanding, this epithelial marker might also contribute to anoikis evasion, survival and establishment of distant metastases in ovarian cancer [48]. Therefore, based on these evidences and considering the decline in colony formation efficiency and in E-cadherin expression (Figure 6A,B), we suggest that *Brassicaceae* extracts and respective ITCs may decrease anchorage-independent cell growth of CRC cells with tumor initiating potential at the expense of the loss of the epithelial phenotype. It seems reasonable that the downregulation of this epithelial marker may induce anoikis, decrease self-renewal potential of HT29 spheroid-derived cells and impair the formation of “second-generation” HT29 spheroids in the semi-solid milieu, in resemblance with previous reports [48,72,73,74]. 

#### 3.2.5. *Brassicaceae* Extracts and ITCs Targeted Differentially Stemness Markers and the Wnt/β-Catenin/TCF7L2 Signaling Axis

To determine whether extracts and respective main bioactive compounds modulate CRC stemness, we examined the effect of extracts and ITCs in *PROM1* (coding for CD133) and *LGR5* expression using the EC_50_ value of each samples, as described previously (Section 3.2.2). *PROM1* and *LGR5* expression have been extensively correlated with CRC stemness, metastasis and chemoresistance. Indeed, it has been reported a positive correlation between the content of CD133^+^CXCR4^+^ cells and the onset of liver metastasis compared to primary tumor in CRC patients, which indicates an enrichment of colorectal CSCs and pinpoints their role in metastasis to a secondary tumor site [10]. Similarly, *LGR5* expression has been implicated in CRC metastasis driven by stem cell-like cells [75], as well as in proliferation, invasion, lymph node metastasis and distant metastasis of CRC as shown in a panel of CRC patient tissue samples [76].

**Figure 7:**
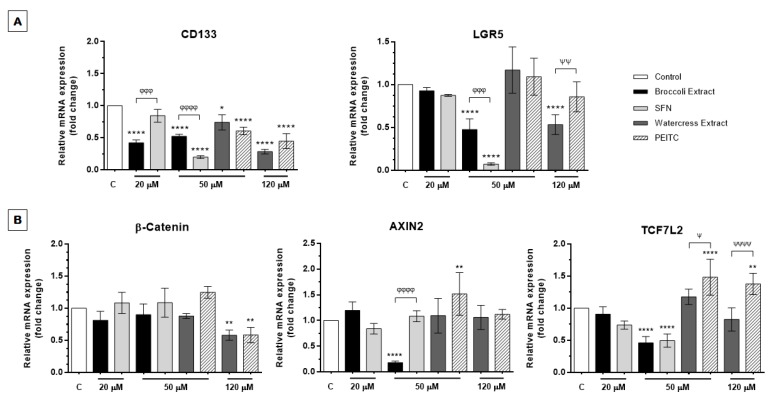
Effects of natural extracts and ITCs in expression of stemness markers (*LGR5* and *PROM1*) and players of the Wnt/β-catenin/TCF7L2 signaling axis (*CTNNB1*, *AXIN2*, *TCF7L2*) related with tumor onset and progression in HT29 spheroids after a 24 h’s treatment; data normalized relatively to treatment with the same % (*v*/*v*) of solvent used for each extract/compound. *GAPDH* was used as endogenous control. (**A**) Analysis of *LGR5* and *PROM1* (coding for CD133) markers expression by qPCR; (**B**) Analysis of *CTNNB1* (coding for β-catenin), *AXIN2* and *TCF7L2* expression by qPCR. For extracts’ EC_50_ values (50 and 120 µM for broccoli and watercress extracts, respectively) results are expressed as mean of two independent experiments performed in triplicate ± SD. For the tested dose bellow the EC_50_ value, results are expressed as mean of one experiment performed at least in triplicate ± SD. * *p <* 0.05, ** *p* < 0.01 and **** *p <* 0.0001 are relative to control; ^φφφ^ *p <* 0.001 and ^φφφφ^ *p <* 0.0001 are relative to broccoli extract; ^Ѱ^ *p <* 0.05, ^ѰѰ^ *p <* 0.01 and ^ѰѰѰѰ^ *p <* 0.0001 are relative to watercress extract.

As shown in Figure 7A, watercress extract and PEITC induced *PROM1* downregulation in a dose-dependent manner, with watercress extract presenting a more pronounced effect with dose increase from 50 µM (*p <* 0.05) to 120 µM (*p <* 0.0001). The same trend was observed for treatment with SFN at 20 µM (not statistically significant) and 50 µM (*p <* 0.0001). However, this tendency was not applied to broccoli extract since this phytochemical amalgam immediately induced *PROM1* downregulation at approximately half of the EC_50_ value (20 µM), an effect that was maintained even when raising treatment dose to 50 µM (*p <* 0.0001). In contrast, the same behavior was not observed for *LGR5* expression. Our results demonstrated that, although broccoli extract and SFN modulated *PROM1* and *LGR5* expression, the extract appeared to be less effective than SFN with dose increase (*p <* 0.0001 and *p <* 0.001, for *PROM1* and *LGR5* expression, respectively). Inversely, watercress extract presented higher effect (*p <* 0.0001) on *LGR5* downregulation compared to the absent PEITC effect. This might be probably due to the presence of other compound(s) in broccoli and watercress extracts that may result in an antagonistic or additive/synergistic effect, respectively, with the corresponding ITCs.

Moreover, although tested under different doses and in different cellular contexts, the downregulation of these markers also correlated with impaired formation of second-generation spheroids under anchorage-independent cell growth conditions (Figure 6). In the case of watercress extract and PEITC the downregulation of stemness markers also correlated with decreased ALDH1 activity (Figure 5A). Altogether, we suggest that both extracts have the capacity to target the colorectal CSC subpopulation and to decrease the self-renewal and metastatic potential of CRC cells. In the case of PEITC and watercress extract their anticancer effects are also fostered by their capacity to decrease ALDH1-mediated drug resistance.

These results assume great importance considering the role of these CSC markers in proliferation and invasion of CRC, as well as with self-renewal and expression of other CSC-related markers [77] and the effect of silencing CSC effectors like LGR5 in HT29 spheroids enriched in CSCs. Indeed, LGR5 silencing was reported to impair colony-formation efficiency in vitro in HT29 and SW480 cells, decreasing proliferation, migration and sphere formation ability in the latter CRC cell line [11].

Therefore, by impairing cell proliferation, migration, anoikis evasion, anchorage-independent cell growth, self-renewal potential and stemness, natural ITC-enriched extracts obtained by high-pressure extraction with supercritical CO_2_ may represent a promising therapeutic strategy for CRC in a context of coadjutant therapies or in alternative to conventional chemotherapeutic drugs, since they target crucial aspects of tumor progression and the CSC-like phenotype that are negligently targeted under the conventional methods that often result in tumor recurrence [7,8]. 

Lastly, we examined the expression of *CTNNB1* (β-catenin), *AXIN2* and *TCF7L2* upon treatment with extracts and ITCs to explore if these phytochemicals also influence the Wnt/β-catenin/TCF7L2 signaling axis, one of the major signaling pathways in CRC carcinogenesis and in invasive and stemness behavior [2]. Our results showed that watercress extract and PEITC promoted a significant downregulation of β-catenin expression with dose increase (*p <* 0.01), whereas no significant differences were observed for broccoli extract and SFN (Figure 7B). In the context of tumor onset, using patient-derived CSCs cultured as spheroids, Vermeulen and colleagues have linked colorectal CSCs homeostasis to Wnt signaling activity and tumor microenvironment, in which stromal myofibroblasts-bearing regions presented higher Wnt signaling activity. In vivo experiments demonstrated that, when inoculated in mice, these CSCs with increased Wnt signaling presented a higher tumor-formation efficiency compared to Wnt^low^ cells [78]. Also, Wnt/β-catenin signaling pathway has shown to modulate growth and maintenance of CSC-enrich HCT116 colonospheres, in which increased levels of β-catenin were related to the induction of TCF family transcription factors-mediated transcriptional activation of growth-related genes [79]. Considering this, our findings suggest that, at least watercress extract and PEITC may exert a promising anticancer effect in colorectal CSC targeting, by modulating β-catenin expression and, hence, CRC progression.

Regarding *AXIN2*, its overexpression correlated with deregulated Wnt signaling pathway has been found in a panel of several CRC cell lines [80]. Indeed, *AXIN2* overexpression has been associated with a pro-oncogenic role in CRC, failing the suppression of the β-catenin/TCF activity and eliciting the acquisition of a mesenchymal phenotype that drives tissue invasion, while *AXIN2* downregulation abrogates this phenotype in vivo [81]. Our results demonstrated that, in general, treatment with extracts and ITCs did not influence significantly *AXIN2* expression with dose increase, suggesting that, eventually, their role in Wnt signaling axis may not be mediated via *AXIN2*. Interestingly, broccoli extract induced a significant *AXIN2* downregulation in a dose-dependent manner suggesting that, contrary to SFN at the same dose, this extract may counteract CRC cells’ invasiveness probably due to a possible additive and/or synergic effect between SFN and minor ITCs or fatty acid derivatives. 

Results also showed that treatment of HT29 spheroids with SFN and broccoli extract induced *TCF7L2* downregulation in a dose-dependent manner (Figure 7B). As an exception, PEITC appeared to induce the opposite effect starting at the lowest dose tested (≈½ EC_50_), promoting a slight increase in *TCF7L2* expression. To note, the role of *TCF7L2* as a tumor inducer [82] or tumor suppressor [83,84] in CRC has been discussed. Considering a tumor suppressor role for *TCF7L2*, it is plausible to assume that PEITC appears to be more effective in targeting CRC tumorigenesis even at the lowest dose. However, considering previous findings that *TCF7L2* role might be cell-type specific, presenting differences between different cell lines, our results need further valuations to explore *TCF7L2* intervention in CSC-related tumorigenesis in HT29 spheroids with heterogenous cell subpopulations.

It is important to highlight that watercress and broccoli extracts may target the Wnt signaling pathway by modulating the expression of different Wnt signaling players. Indeed, watercress extract may regulate Wnt signaling through the downregulation of β-catenin, whereas broccoli extract may modulate this signaling pathway by reducing *TCF7L2* and *AXIN2* expression hence, leading to downregulation of Wnt/TCF7L2 signaling, as corroborated by p21 upregulation (p21 is upregulated by MYC downregulation upon Wnt signaling inhibition) (Figure 4B) [85].

Overall, our results suggest that these ITCs and ITCs-enriched extracts display a promising role in targeting CSC and Wnt/β-catenin signaling pathway, although the effect of broccoli extract/SFN and watercress extract/PEITC may differ regarding specific mechanisms and cell-contexts. Nevertheless, further studies should be performed in HT29 spheroids to explore other putative CSC markers, as well as other targets of signaling pathways inherent to CSCs.

Currently, in completed and on-going clinical trials in cancer cases, these bioactive compounds are administrated orally in the form of watercress and broccoli juices/extracts or capsules, for example by consuming the equivalent to 980–1225 µmol PEITC or 200 µmol SFN in a daily basis (NCT00968461; NCT03034603; NCT01228084) [86]. However, as already mentioned, the biological uptake/absorption of ITCs and the attainment of an effective therapeutic dose to eradicate CSCs at the target site can depend on several factors. The doses tested in our 3D CRC cell model varied between 1–120 µM of PEITC and 1–50 µM of SFN, which are well below the values used in clinical trials or in vivo models. Although some of these concentrations exceed the cytotoxicity IC_50_ values obtained in the Caco-2 intestinal barrier model (Table S1, Supplementary Material) further studies should be carried out in animal models aiming at determining the safety and bioactive doses of ITC-enriched supercritical fluid extracts, as well as their side-effects, and, according to the results, design the most suitable administration route of these nutraceuticals. In this context, in a near future, it would be reasonable to circumvent cytotoxicity issues by developing nanocarriers coupled with targeting moieties directed to tumor site, in order to achieve a selective delivery (orally or intravenously) of phytochemicals encapsulated in nanoparticulate delivery systems [87,88].

## 4. Conclusions

To our knowledge this is the first report recognizing the effects of supercritical fluid extracts enriched in PEITC and SFN in modulating CSCs, chemoresistance and metastatic potential of CRC cells in a 3D cellular scenario that best resembles with the tumor in vivo. Overall, both extracts proved to impair cell proliferation in HT29 spheroids arresting cell cycle at G_2_/M checkpoint, probably due to DNA damage. Moreover, both extracts also decreased cell self-renewal and in vitro colony formation in anchorage-independent cell growth conditions, which correlated with loss of cell-cell adhesion (E-cadherin downregulation), suggesting the impairment of the metastatic skills of stem and circulating tumor cells that may colonize a secondary target site. Moreover, *Brassicaceae* extracts, especially watercress extract, demonstrated also the ability to target the CSC subpopulation and to reduce the ALDH1-mediated chemoresistance of these cells by decreasing the expression of stemness markers (*LGR5* and *PROM1*) and by impairing ALDH1 activity, as well as the ability to target CRC progression by targeting the Wnt/β-catenin/TCF7L2 signaling pathway. 

Altogether, our data strongly support the chemotherapeutic potential of ITC-enriched extracts in CRC therapy by targeting critical aspects of tumor progression and tumor relapse, namely CRC stem and tumor initiating cells, as well as their metastatic potential. This study unveils an exciting start-point to open the door for new therapeutic strategies resorting to the use of natural extracts for CRC treatment, while avoiding the limitations of the current therapies, as well as the use of standard bioactive compounds chemically produced under toxic procedures. 

## Supplementary Materials

The following are available online at http://www.mdpi.com/2072-6643/9/4/368/s1, Table S1: IC_50_ values relative to the cytotoxic effects of broccoli extract, SFN, watercress extract and PEITC in Caco-2 cells, Figure S1: Morphology of HT29 2D and 3D cell models assessed by light microscopy, Figure S2: Phenotypical characterization of HT29 2D and 3D cell models regarding ALDH1 activity and in vitro colony formation efficiency, Figure S3: Analysis of *ABCB1* (coding for P-gp) expression by qPCR in HT29 spheroids treated with EC_50_ values of both natural extracts and ITCs at equal concentrations for 24 h, Figure S4: Average colony size (in µm) of 2nd generation HT29 spheroids in semisolid agar after 2weeks of treatment with SFN and broccoli extract (with 1, 3 and 5 µM). Reference [17] is cited in the supplementary materials
