# Supplementary material for: Targeting Colorectal Cancer Proliferation, Stemness and Metastatic Potential Using Brassicaceae Extracts Enriched in Isothiocyanates: A 3D Cell Model-Based Study
Source: Nutrients. 2017 Apr 10;9(4):368. doi: 10.3390/nu9040368 (PMC5409707; doi:10.3390/nu9040368)
Supplement: Supplementary file 1 [file nutrients-09-00368-s001.docx]

**Supplementary Materials**

Targeting Colorectal Cancer Proliferation, Stemness and Metastatic Potential Using *Brassicaceae* Extracts Enriched in Isothiocyanates: A 3D Cell Model-Based Study

Lucília P. Pereira, Patrícia Silva, Marlene Duarte, Liliana Rodrigues, Catarina M. M. Duarte, Cristina Albuquerque and Ana Teresa Serra

**Table S1**. IC_50_ values relative to the cytotoxic effects of broccoli extract, SFN, watercress extract and PEITC in Caco-2 cells.

| Extract/ITC | Cytotoxicity Assay (Caco-2) |
| --- | --- |
|  | IC_50_ (in µM of ITC) |
| Broccoli Extract | 35.7 ± 1.2 |
| SFN | 64.9 ± 2.4 |
| Watercress Extract | 67.0 ± 4.1 [1] |
| PEITC | 79.1 ± 6.0 [1] |


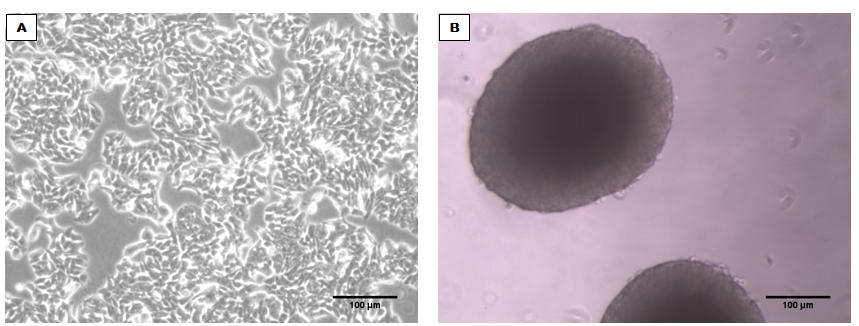


**Figure S1.** Morphology of HT29 2D and 3D cell models assessed by light microscopy. **(A)** HT29 cell monolayer (2D cell model). **(B)** HT29 spheroids collected at 7th day of spinner vessel culture (3D cell model), encompassing a high cell density (about 15.000-20.000 cells/spheroid). Scale bar of 100 µm.


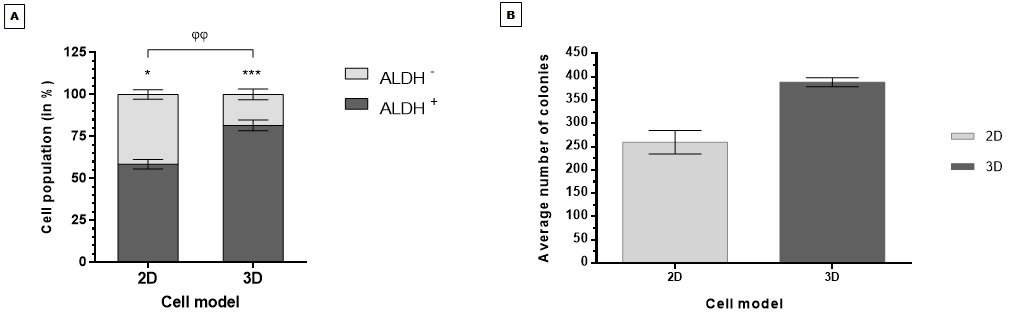


**Figure S2.** Phenotypical characterization of HT29 2D and 3D cell models regarding ALDH1 activity and in vitro colony formation efficiency. **(A)** HT29 spheroids are enriched in ALDH^+^ cells compared to 2D model, as shown by the higher ALDH1 activity in the 3D cell model. Results are expressed as mean of at least two independent experiments performed in duplicate ± SD. *p-value < 0.05 and ***p-value < 0.001 are relative to the overall population of the respective cell model; ^φφ^p-value < 0.01 relative to 2D cell model. **(B)** HT29 spheroids presented increased potential to evade anoikis and to proliferate under anchorage-independent conditions, compared to 2D model, as shown by the highest colony formation efficiency. Results are expressed as mean of at least two independent experiments performed in duplicate ± SD.

**Figure S3.** Analysis of *ABCB1* (coding for P-gp) expression by qPCR in HT29 spheroids treated with EC_50_ values of both natural extracts and ITCs at equal concentrations for 24h; data normalized relatively to treatment with the same % (v/v) of solvent used for each extract/compound. *GAPDH* was used as endogenous control. Neither extracts or ITCs induced significant variations in P-gp expression at the EC_50_ of both extracts. Results are expressed as mean of one experiment performed in triplicate ± SD.


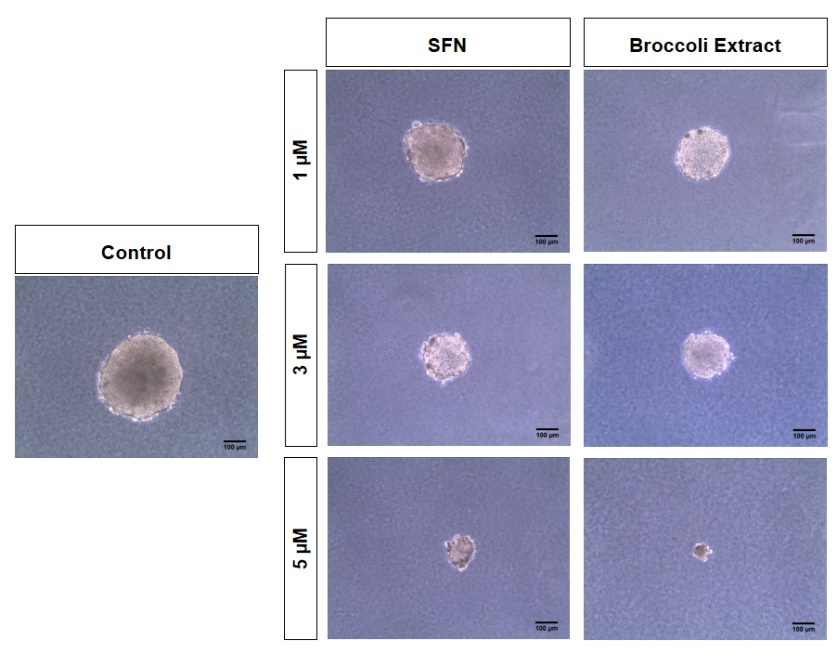


**Figure S4.** Average colony size (in µm) of 2nd generation HT29 spheroids in semisolid agar after 2 weeks of treatment with SFN and broccoli extract (with 1, 3 and 5 µM). Soft agar assay reveled that both phytochemicals reduced the mean diameter of colonies in a dose-dependent manner under anchorage-independent independent conditions. Scale bar = 100 µm.

References

1. Rodrigues, L.; Silva, I.; Poejo, J.; Serra, A.T.; Matias, A.A.; Simplicio, A.L.; Bronze, M.R.; Duarte, C.M.M. Recovery of antioxidant and antiproliferative compounds from watercress using pressurized fluid extraction. *RSC Adv.* **2016**, *6*, 30905–30918.
